# Supplementary material for: Sorption of Monothioarsenate to the Natural Sediments and Its Competition with Arsenite and Arsenate
Source: Int J Environ Res Public Health. 2021 Dec 6;18(23):12839. doi: 10.3390/ijerph182312839 (PMC8657673; doi:10.3390/ijerph182312839)
Supplement: Supplementary file 1 [file ijerph-18-12839-s001.zip › ijerph-1466992-SI.pdf]

Supplementary Materials

Title: Sorption of monothioarsenate to the natural sediments and its competition with arsenite and arsenate

Huimei Shan [1,2,*], Jinxian Zhang [1,2], Sanxi Peng [3], Hongbin Zhan [4,*] and Danxue Liao [1,2]

1 Guangxi Key Laboratory of Environmental Pollution Control Theory and Technology, Guilin University of Technology, Guilin 541004, China
2 College of Environmental Science and Engineering, Guilin University of Technology, Guilin 541004, China
3 College of Earth Science, Guilin University of Technology, Guilin 541004, China
4 Department of Geology Geophysics, Texas A＆M University, 77843, USA

* Correspondence: shanhuimei@glut.edu.cn (H.S.); zhan@geos.tamu.edu (H.Z.)

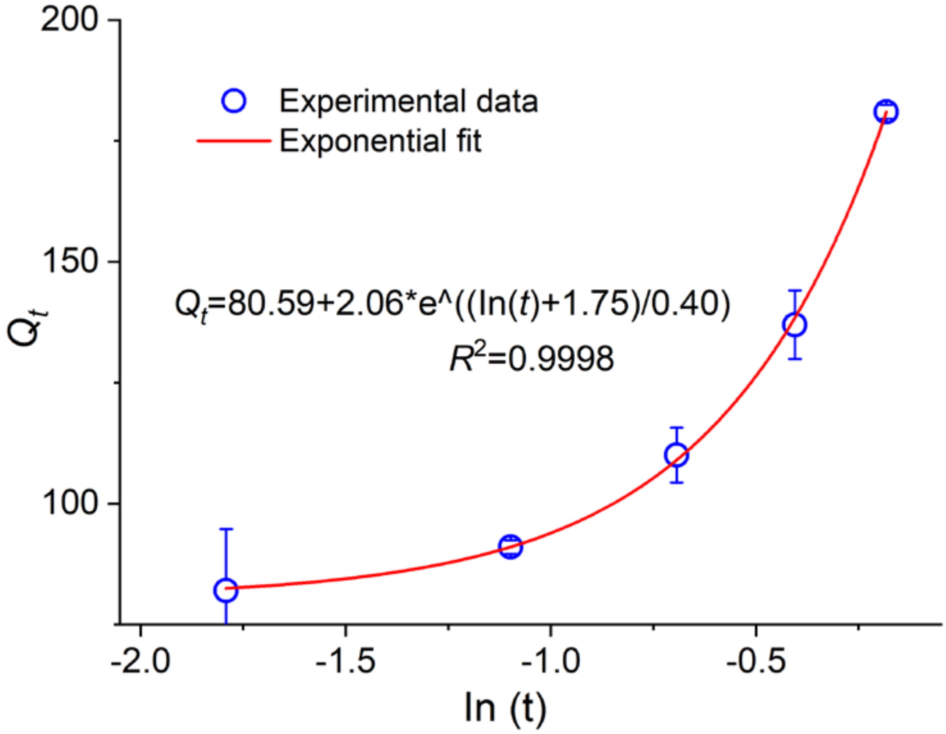

Figure S1. Exponential fitting curves of the relationship between In(t) and Q_t for 0.667 mmol/L $MTAs^V$ adsorption within 50 min.

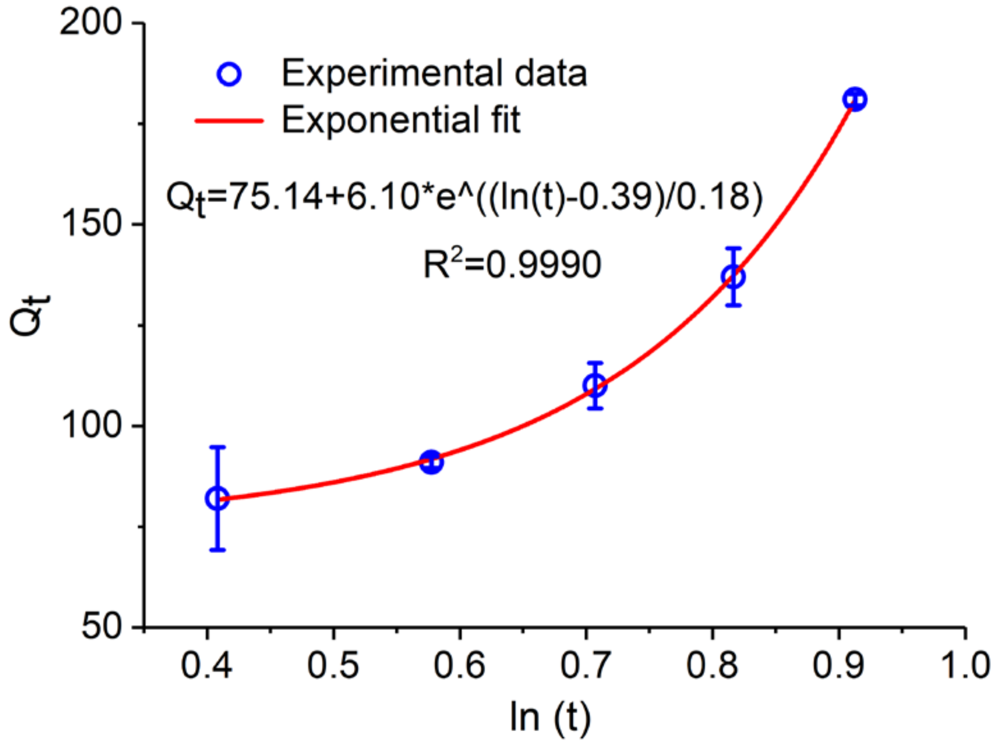

Figure S2. Exponential fitting curves of the relationship between $t^{0.5}$ and Q_t for 0.667 mmol/L $MTAs^V$ adsorption within 50 min.

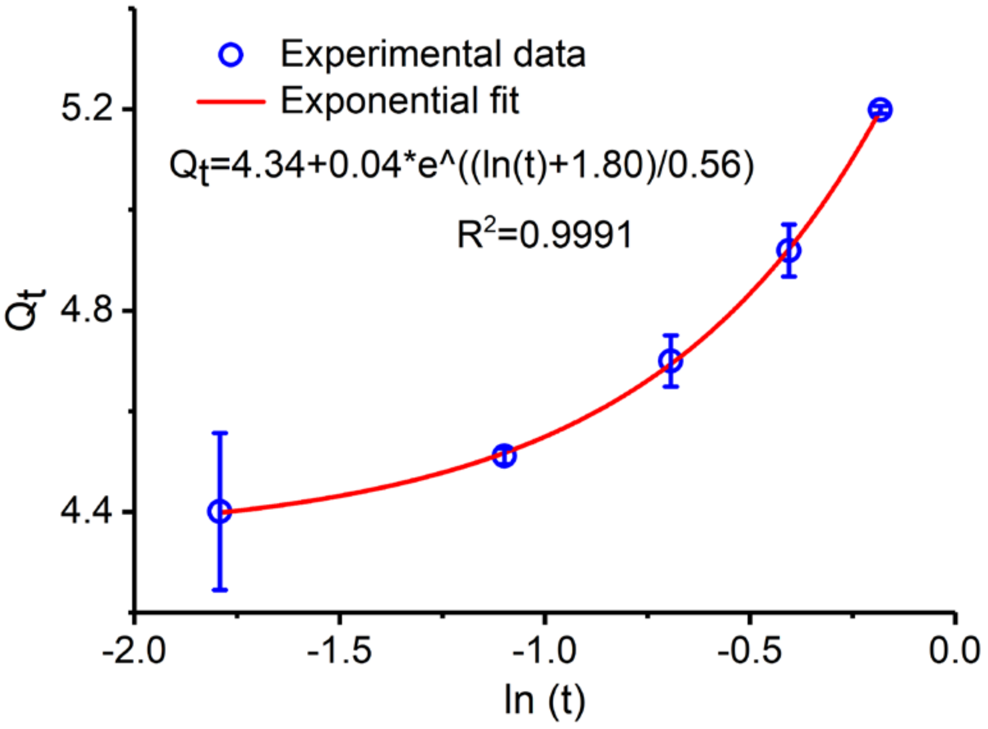

Figure S3. Exponential fitting curves of the relationship between In(t) and In(Q_t) for 0.667 mmol/L $MTAs^V$ adsorption within 50 min.
